# Local Electronic Correlation in Multicomponent Møller–Plesset Perturbation Theory

**DOI:** 10.1021/acs.jctc.4c01059

**Published:** 2024-11-08

**Authors:** Lukas Hasecke, Ricardo A. Mata

**Affiliations:** Institute of Physical Chemistry, University of Göttingen, Tammannstrasse 6, 37077 Göttingen, Germany

## Abstract

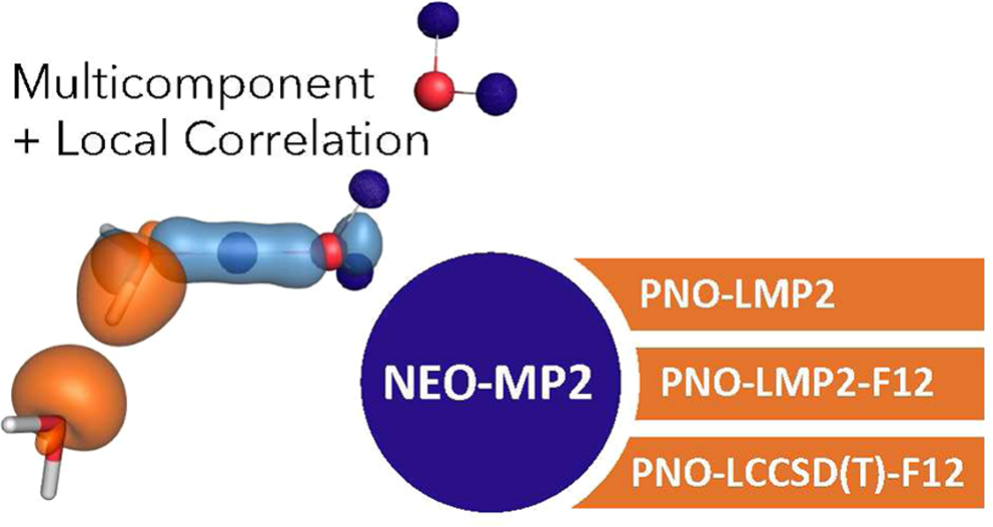

We present in this contribution the first application of local correlation in the context of multicomponent methods. Multicomponent approaches allow for the targeted simulation of electrons together with other Fermions (most commonly protons) as quantum particles. These methods have become increasingly popular over the last years, particularly for the description of nuclear quantum effects (in strong hydrogen bonds, proton tunneling, and many more). However, most implementations are still based on canonical formulations of wave function theory, which we know for decades to be computationally inefficient for capturing dynamical correlation effects. Local correlation approaches, particularly with the use of pair natural orbitals (PNOs), enable asymptotically linear scaling of computational costs with very little impact on the overall accuracy. In this context, the efficient use of density fitting approximations in the integral calculation proves essential. We start by discussing our implementation of density-fitted NEO-MP2 and NEO-PNO-LMP2, upgrading the electronic correlation treatment up to PNO local coupled cluster level of theory. Several challenging examples are provided to benchmark the method in terms of accuracy as well as computational cost scaling. Following appropriate protocols, anharmonic corrections to localized X-H stretches can be applied routinely with little computational overhead.

## Introduction

Nuclear quantum effects (NQEs) play an important role in quantum chemistry, becoming particularly significant in scenarios such as conical intersections, molecular vibrations, and low-temperature atomic coherence and exchange effects.^1^ This is especially true for light nuclei such as hydrogen, where even at room temperature NQEs cannot be overlooked. Traditional quantum chemical calculations often rely on the Born–Oppenheimer (BO) approximation to separate the electronic and nuclear degrees of freedom. While this allows for classical propagation of nuclei on the electronic potential energy surface or the use of approximations to extract dynamic nuclear information, it fails to capture the substantial coupling between nuclear and electronic wave functions that directly influence chemical phenomena.

To address these challenges, various methodologies have been developed. Multicomponent methods stand out, offering a way to handle nuclei and electrons quantum mechanically on the same level, thus inherently including anharmonic effects, nuclear delocalization, zero-point vibrational energies (ZPVE), and quantum tunneling. Historical contributions from Thomas^2,3^ and pioneering works by Parr and Gross^4,5^ have led to the development of several branches and implementations of this multicomponent treatment, such as the dynamical extended molecular orbital (DEMO), nuclear orbital molecular orbital (NOMO), multicomponent molecular orbital (MCMO), electronic and nuclear molecular orbitals (ENMO) approaches,^6−11^ as well as the most commonly discussed framework today, the nuclear electronic orbital (NEO) approach.^12^ We recognize that the similarities between the different implementations are greater in number than the differences, but in the lack of a consensual nomenclature, we opt for the use of the NEO acronym in the following text.

In a previous work, we introduced a local density fitting NEO-Hartree-Fock (LDF-NEO-HF) program, which exhibits a small computational footprint, allowing for the routine calculation of several quantum protons in extended systems on a regular workstation.^13^ Furthermore, we introduced a rather simple protocol to determine on-the-fly the positions of quantum nuclei. The latter works by updating the nuclei positions to the respective nuclear orbital charge centroids during the self-consistent field (SCF) cycles.^14^ This potentially allows for the use of smaller nuclear basis sets, as the nuclear densities are aligned with the nuclear basis centers. This can also be enforced by the constrained NEO ansatz.^15^ In these earlier works, however, we failed to include dynamic correlation effects. In particular, the electron–proton correlation is deemed significant for obtaining nuclear densities and energetics, albeit their relative impact on NEO calculations is still disputed.^16^ In general, one can argue that the question of correlation in multicomponent approaches is still today a very current topic.^17^

In the following section, we introduce the basics of second-order Møller–Plesset perturbation theory within the NEO framework, as well as a short overview of density fitting and local correlation treatments.

## Methods

### NEO-MP2

The original derivation of the NEO-MP2 method was done by Swalina et al.^18^ using the reference Hamiltonian from NEO-Hartree-Fock theory
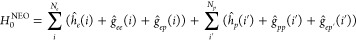
1where *N*_e_ is the number of electrons and *N*_p_ is the number of quantum mechanically treated nuclei, which are referred to by primed indices. The one-particle Hamiltonians for the respective system are denoted as *ĥ* and the two-particle Coulomb exchange operators are defined as
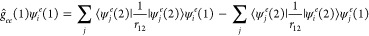
2
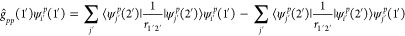
3where ψ denotes spin orbitals and the sum {*j*, *j*′} includes the respective occupied spin orbitals of the electrons and protons. The electron–proton Coulomb operators are defined as
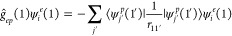
4
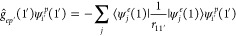
5By employing Rayleigh–Schrödinger perturbation theory the NEO-MP2 energy expression for the multicomponent electronic–nuclear wave function is defined as
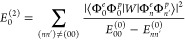
6where |Φ_0_^*e*^Φ_0_^*p*^⟩ is the zeroth-order wave function of the ground state and |Φ_*n*_^*e*^Φ_*n*′_^*p*^⟩ denotes the excited states with the corresponding sum of the orbital energies *E*_*nn*′_^(0)^. The perturbation *W* = *W*_*ee*_ + *W*_*pp*_ + *W*_*ep*_ consists of the individual electron–electron, proton–proton, and electron–proton perturbations which are given as
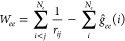
7
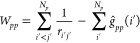
8
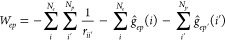
9By integrating out the spin, taking electronic spatial orbitals as doubly occupied and nuclear orbitals as singly occupied, the following equations are used to obtain the individual contributions of the NEO-MP2 energy
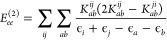
10
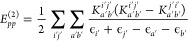
11
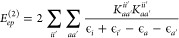
12where {*i*, *j*} refer to occupied spatial orbitals, {*a*, *b*} refer to virtual spatial orbitals, ϵ are the respective orbital energies, and *K*_*ab*_^*ij*^ = (*ia*|*jb*) are the two-particle 4-index integrals.

### DF-NEO-MP2

The two-particle 4-index integrals can also be defined as the interaction of two orbital-product densities
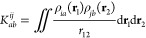
13The individual one-particle densities ρ_*ia*_(**r**) = ϕ_*i*_(**r**)ϕ_*a*_(**r**) can be approximated as an expansion
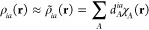
14in the auxiliary fitting basis functions {χ_*A*_}.^19^ Several strategies to determine the expansion coefficients can be employed, but in our case this is done by minimizing the least-squares error of the electric field, commonly referred to as robust density fitting.^20^ Thereby, the coefficients are obtained by the minimization of the positive definite functional
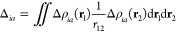
15where Δ*ρ*_*ia*_(**r**) = ρ_*ia*_(**r**) – ρ̃_*ia*_(**r**) refers to the difference between the exact ρ_*ia*_ and the approximated ρ̃_*ia*_ one-particle density.^21^ As a result, the coefficients can be obtained as
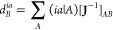
16where *J*_*AB*_ is the Coulomb interaction between the auxiliary fitting basis functions and
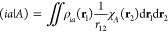
17are the two-particle 3-index integrals formed by the one-particle density and the auxiliary fitting basis functions. The obtained coefficients can now be employed to approximate the four-center two-particle integrals as
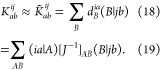
This procedure is analogously done for the nuclear two-particle 4-index *K*_*a*′*b*′_^*i*′*j*′^ integrals. However, for the two-particle 4-index integrals built from electrons and nuclei *K*_*aa*′_^*ii*′^ the density fitting can be employed within the electronic or nuclear auxiliary basis set. This was already further explored by the group of Hammes-Schiffer.^22,23^ It was found that by employing the density fitting approximation with the nuclear density fitting basis, the overall error in forming those integrals can be significantly reduced. Therefore, we also employed the nuclear density fitting within the nuclear part of the mixed interactions between electrons and nuclei. Moreover, we unify both the electronic and nuclear auxiliary fitting basis sets since this avoids the recomputation and repeated transformation of the 3-index integrals within the electronic part. This approach reduces the overall computational costs, especially if the electronic subsystem exceeds the nuclear subsystem, which we have discussed previously in the construction of our LDF-NEO-RHF program.^13^ Overall, the asymptotic scaling with the fifth order is not reduced by the density fitting approximations within the DF-NEO-MP2 program.^19^ However, the density fitting leads to a low prefactor of the nominally quintic scaling which leads to a significant computational speed-up for small to midsized molecules as we will demonstrate later.

### NEO-PNO-LMP2

With the aim to make multicomponent wave function-based correlation methods accessible for large systems, further approximations must be applied to lower the computational scaling. This can, for example, be achieved by localizing the delocalized canonical orbitals. This localization can be used to lower the quintic scaling of the canonical DF-NEO-MP2 to an asymptotic linear scaling in the electron terms.^19,24^ In the context of this work, we make use of the pair natural orbital (PNO) PNO-LMP2 approach as proposed by Werner and co-workers and apply it to the electron–electron correlation.^24^ We briefly summarize the methodology; further details and associated works can be found in the original reference. The MP2 correlation energy can be constructed as the sum of orbital pair energies, with
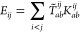
20whereby *T̃*_*ab*_^*ij*^ = 2*T*_*ab*_^*ij*^ – *T*_*ba*_^*ij*^ are the contravariant amplitudes, constructed from the *T*_*ab*_^*ij*^ wave function amplitudes, which weight excitations from a pair of occupied orbitals *ij* to a pair of virtual orbitals *ab*. When using canonical orbitals (see eq 10), it is difficult to reduce the computational effort associated with the electron repulsion integrals (ERIs) *K*_*ab*_^*ij*^ (see eq 13). However, if the occupied orbitals are unitarily transformed to localized molecular orbitals (LMOs), only a subset of pair energies are required. Those can be obtained from various localization procedures like Foster-Boys, Pipek-Mezey, or as intrinsic bond orbitals (IBOs).^25−27^ In such a representation, only when an occupied electronic orbital ϕ_*i*_ is in the vicinity of ϕ_*j*_ does one need to (accurately) calculate its value, as the dynamical electronic correlation rapidly decays with the interelectronic distance.

Further savings can be achieved by truncating the virtual space, as well. This requires more advanced treatments since not only the virtual space is much harder to localize, one needs to build a robust selection of orbitals ϕ_*a*_ and ϕ_*b*_ for each *ij* pair. For this purpose, one makes use of PNOs, which are obtained by diagonalizing (approximate) external MP2 pair density matrices
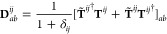
21The natural occupation number associated with each PNO is used to build pair-specific domains [*ij*]_PNO_. The number of ERIs needed is thereby immensely reduced. In order to provide a compact localization of the virtual space a hierarchical treatment of projected atomic orbitals, orbital-specific virtuals, and pair natural orbitals, as introduced by the group of Werner, is employed.^24,28^ Moreover, multipole approximations for distant pairs can be used to further reduce the scaling.^24^

There are several reasons that such a local scheme (albeit possible) is not used for the terms involving nuclei. Foremost, the calculation of the nuclear–nuclear and electronic–nuclear correlation is, for all of the examples provided in this paper, a minute fraction of the computational cost as we demonstrate later on. In all cases, computation of the electronic correlation overwhelmingly dominates the timings. Notwithstanding, and as mentioned previously in the text, we significantly decreased the computational demand of the electronic–nuclear 4-center integrals by employing local approximations. The latter quantities are calculated using the nuclear fitting basis, whereby only contributions from the electrons in the proximity of the nuclei are accounted for. Although it might seem like a harsh approximation, given the local nature of the electron–proton interaction, this turns out to be a very robust approach.^22,23^

### Composite NEO Correlation Methods

In the NEO-MP2 framework, the amplitudes for each interaction type are decoupled. This allows one to treat specific terms, for example, the electron–electron correlation, on a different computational level than the electronic–nuclear and nuclear–nuclear correlation. The level of theory for the electronic correlation, which is a particularly sensitive quantity, can be improved without any change in the other correlation terms. Such modular approaches to the correlation energy are not new within the NEO framework and are regularly employed for NEO-DFT. Thereby, it is a common approximation to neglect the nuclear–nuclear correlation due to the low impact on the overall correlation energy.^29−31^ Moreover, sometimes even the electronic–nuclear correlation is neglected and only the electron–electron correlation is accounted for.^16,32^ In order to make clear that the nuclear–nuclear and nuclear electronic correlations are handled at the MP2 level, but the electronic–electronic correlation is described at a higher level of theory X, we denote the composite method as NEO(MP2)-X.

Making use of the Molpro program package capabilities, it is possible, for example, to include explicit correlation for the electron–electron interactions (NEO-PNO-LMP2-F12) in order to minimize the error of an incomplete basis set and to reduce the domain error.^33,34^ The computation of the electronic correlation energy can be further extended, for example, up to a local coupled cluster with singles and doubles excitations and perturbative triples (PNO-LCCSD(T) and PNO-LCCSD(T)-F12). This involves the approximation that the electronic and nuclear amplitudes remain decoupled, following the idea of the NEO-DFT implementations where contributions from electron–electron and nuclear–nuclear interactions are treated independently from each other and are also not directly considered for the electronic–nuclear correlation.^16,29−32,35−38^ The efficient implementations of both aforementioned programs have been described in detail by the group of Werner.^24,34,39,40^

## Computational Details

Throughout this work, all NEO calculations are carried out with the PB4-F2 nuclear basis set together with the even-tempered 10s10p10d10f fitting basis set with exponents ranging from 2√2 to 64 using Molpro.^13,41,42^ The cc-pVTZ electronic basis set for the benchmark calculations of our DF-NEO-MP2 and NEO-PNO-LMP2 implementations was employed together with the cc-pVXZ-JKFIT density fitting basis set at the X = T triple-ζ and X = Q quadruple-ζ level.^43,44^ The reference wave function was built by DF-NEO-RHF for the DF-NEO-MP2 method and LDF-NEO-RHF for the NEO-PNO-LMP2 method. Thereby, we set a threshold of 10^–8^ au for the energy difference within the electronic and nuclear SCF computations, the difference in the density between iterations, and the gradient of the respective nuclear and electronic subiterations. The overall energy difference in the NEO-RHF iterations was set to a threshold of 10^–7^ Hartree for all systems besides the DNA strands where the threshold was set to 10^–6^ Hartree. All SCF computations employ the direct inversion in the iterative subspace starting after the first iteration with a maximum of 10 Fock matrices as a basis to extrapolate.^45,46^ All results following the benchmark calculations are obtained for the wave function from DF-NEO-RHF with the settings mentioned before. However, in order to provide the most suitable basis set for the explicitly correlated methods the electronic basis set was changed to cc-pVTZ-F12 with the cc-pVQZ-JKFIT density fitting basis for the Fock and the exchange matrices as well as the complementary auxiliary basis set for the resolution of the identity and the cc-pVQZ-MP2FIT density fitting basis set.^44,47,48^ The presented F12B energies were obtained by employing the 3*A(LOC,FIX) ansatz with very tight domain settings and corrected by the complementary auxiliary basis set singles correction together with the scaling of the perturbative triples.^34,39,40,49^ The water tetramers and methanol-furan complexes were optimized with Gaussian16^100^ employing the def2-TZVPP basis set with very tight SCF settings, tight optimization thresholds and a superfine grid utilizing B2PLYP-D3(BJ) and additionally for the methanol-furan complexes DSDPBEP86-D3(BJ) as well as B3LYP-D3(BJ).^50−53^ Energies and frequencies shown in this work were obtained with the same settings. In the case of the proton-bound dimer of hydrogen sulfate and formate, the structures were taken from Thomas et al.^54^ According to their original work, the energies and frequencies were recomputed by utilizing the aforementioned settings and the aug-cc-pVTZ basis set. All computations include the D3 dispersion correction with Becke-Johnson damping.^52,56^ The DNA model was constructed with Avogadro 1.2.0.^58^ All nuclear densities shown are at a 0.01 σ contour level generated with the PyMOL 2.5.2 program.^59^ The DF-NEO-MP2 benchmark calculations were computed on a commercially available Intel Xeon Gold 6226R 2.90 GHz CPU with 8 threads, and the NEO-PNO-LMP2 benchmark calculations utilized a commercially available Intel Xeon Gold 6342 2.80 GHz CPU with 8 threads.

## Results and Discussion

### DF-NEO-MP2

**Figure 1:**
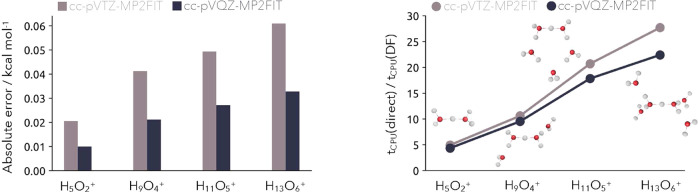
Left: Absolute error of the DF-NEO-MP2 method computed with the cc-pVTZ-MP2FIT (light gray) and cc-pVQZ-MP2FIT (dark gray) density fitting basis set in comparison to the integral-direct NEO-MP2 results for size increasing protonated water clusters. Right: Computational speed-up was achieved by the DF-NEO-MP2 method with the two fitting basis sets in comparison to the integral-direct NEO-MP2 version.

With the aim to benchmark the accuracy and computational performance of the DF-NEO-MP2 implementation, we compare the results to an integral-direct NEO-MP2 implementation and generally compare the achieved accuracy with previously reported density fitting approximations for NEO methods.^23,60^ For that reason, we employ size increasing protonated water clusters as trial systems. These are well-established reference systems for multicomponent methods.^61,62^ Preliminary error analysis of different density fitting methods within the NEO framework was already carried out by the group of Hammes-Schiffer for the density fitting of NEO specific types of integrals (electron–electron, electron–proton, proton–proton) within the DF-NEO-CCSD method, in general for the accuracy of the DF-NEO-MP2 determining proton affinities and for the absolute deviation of the water energy with one and two quantum protons of the DF-NEO-OOMP2 method.^23,60^ However, the overall scaling of the density fitting error with respect to increasing system sizes and the achieved computational performance remains elusive. Therefore, we computed the absolute deviation of the obtained energy from DF-NEO-MP2 obtained with the cc-pVTZ-MP2FIT and cc-pVQZ-MP2FIT density fitting basis sets to a complete integral-direct NEO-MP2 version and compared also the computational effort of these methods for the size increasing protonated water clusters. The results are shown in Figure 1 with the corresponding system information and computational timings provided in Table 1. By increasing the fitting basis set size it is possible to reduce the error from 0.02 to 0.06 kcal mol^–1^ for the cc-pVTZ-MP2FIT set down to 0.01–0.03 kcal mol^–1^ for the cc-pVQZ-MP2FIT set. The overall error with respect to the increasing system size scales rather linearly, and the obtained errors introduced by the density fitting approximation are similar to those observed by Hammes-Schiffer and co-workers. Regarding the computational savings by employing the density fitting approximation, the speed-ups range between factors of 5–28 for the cc-pVTZ-MP2FIT set and only slightly reduced to a factor of 4–22 for the cc-pVQZ-MP2FIT set. The achieved computational speed-up is especially notable since the overall error can be reduced by half when employing the larger cc-pVQZ-MP2FIT and the loss in speed-up compared to the smaller cc-pVTZ-MP2FIT is only minor. The density fitting error of the proton–proton and electron–proton contributions, which depend both on the nuclear density fitting basis set, was also discussed in previously mentioned works from the Hammes-Schiffer group. In our work, we consistently employ the 10s10p10d10f set as a nuclear density fitting basis which leads to an absolute deviation of 4 × 10^–8^ kcal mol^–1^ for the largest protonated water cluster including 13 quantum protons. This is, for all purposes, a negligible error. This high accuracy of the nuclear density fitting can be exploited for the computation of the electron–proton correlation, where the protonic part is treated with the density fitting approximation. This leads to an absolute deviation of 4 × 10^–4^ kcal mol^–1^ for the electron–proton correlation energy of the largest protonated water cluster. This is generally consistent with the error of the proton–proton correlation since the absolute contribution of the electron–proton correlation is 4 orders of magnitude larger than the proton–proton correlation. Although the employed nuclear density fitting basis set seems fairly large, the actual impact of the total computational timing is minor, even for extended systems. This is demonstrated in the next section. Overall, the absolute error of the density fitting approximation within the NEO-MP2 method is governed by the fitting of the electron–electron correlation terms.

**Table 1:** System Information on the Protonated Water Clusters and Corresponding Computational Timings for the Integral-Direct NEO-MP2 as well as the DF-NEO-MP2 Method with the Unified Density Fitting Basis of 10s10p10d10f with cc-pVTZ-MP2FIT and cc-pVQZ-MP2FIT

structures	H_5_O_2_^+^	H_9_O_4_^+^	H_11_O_5_^+^	H_13_O_6_^+^
basis functions (electronic, nuclear)	(130, 185)	(246, 333)	(304, 407)	(362, 481)
total fitting basis functions (cc-pVTZ-MP2FIT)	1112	2034	2495	2956
total fitting basis functions (cc-pVQZ-MP2FIT)	1339	2463	3025	3587
nuclear fitting basis functions	800	1440	1760	2080
occupied orbitals (electronic, nuclear)	(10, 5)	(20, 9)	(25, 11)	(30, 13)
*t*_CPU_ (integral-direct NEO-MP2) /s	25.3	227.8	746.6	1580.1
*t*_CPU_ (DF-NEO-MP2, cc-pVTZ-MP2FIT) /s	5.1	21.5	36.1	57.0
*t*_CPU_ (DF-NEO-MP2, cc-pVQZ-MP2FIT) /s	5.8	23.9	41.9	70.5

### NEO-PNO-LMP2

**Figure 2:**
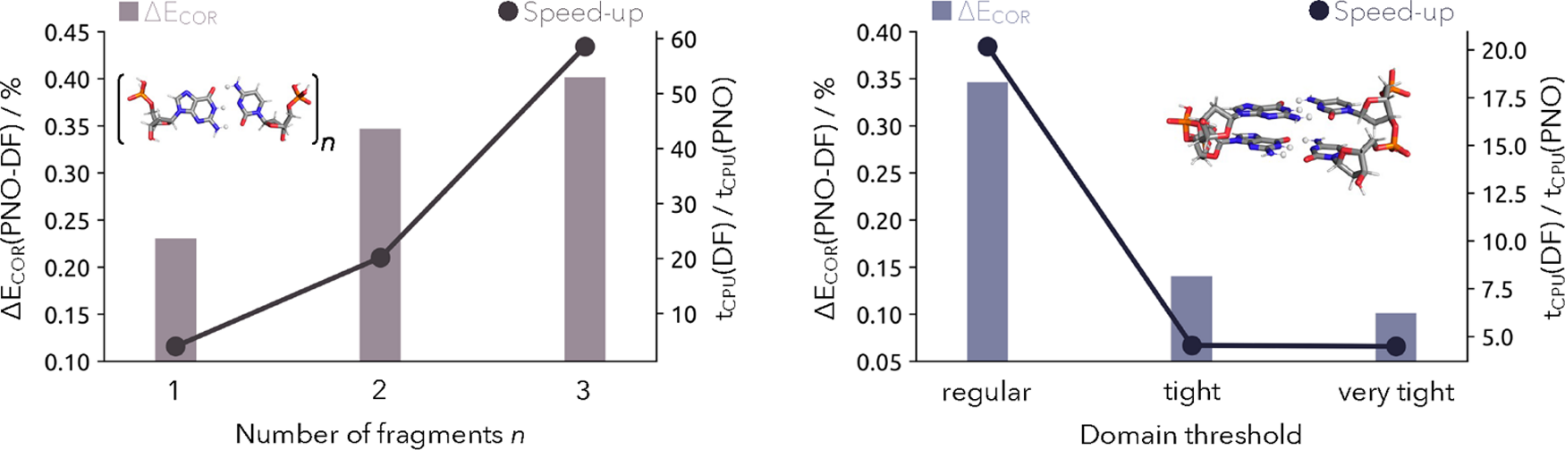
Left: Percentage of the unaccounted correlation energy (light gray) by utilizing the NEO-PNO-LMP2 method in comparison to the DF-NEO-MP2 method together with the achieved computational speed-up (dark gray) for an increasing DNA strand of guanine-cytosine base pairs (regular domains). Right: Percentage of the unaccounted correlation energy (light blue) and achieved computational speed-up (dark blue) of the local NEO-PNO-LMP2 method with increasing domain sizes in comparison to the canonical DF-NEO-MP2 method for two guanine-cytosine base pairs.

While the DF-NEO-MP2 method is excellent both in terms of accuracy as well as computational speed-up for small- to midsize systems, it is itself still computationally prohibitive for large systems. This is due to the asymptotically quintic scaling in the integral assembly. However, this can be significantly reduced through the use of local approximations.^19,24^ As the published work on Born–Oppenheimer-based electronic PNO-LMP2 shows, the localization in PNOs yields a computationally very efficient approach since even a minimal domain size is sufficient to already recover a vast majority of the electronic correlation energy.^24^ However, this approximation was not used before on a reference wave function obtained from a multicomponent method, so it has yet to be demonstrated for this case. Therefore, we employ as test system a DNA fragment built from guanine-cytosine base pairs, one of the most important molecules from biochemistry and consistently size-increasable by adding more base pairs to the strand.^63,64^ At first we compare the achieved accuracy and computational efficiency with the already optimized default settings for PNO-based methods in Molpro in comparison to our canonical DF-NEO-MP2 implementation.^42^ The results of this benchmark are shown in Figure 2 with the corresponding system information and computational timings provided in Table 2. By increasing the number of guanine-cytosine base pairs from one to three the unaccounted correlation energy rises only from 0.23 to 0.40%, so overall the recovered correlation energy is in line with previous benchmarks for the PNO method when employed in a multicomponent context.^24^ By tightening the threshold for the local density fitting and the RI approximations as well as for the domain approximations, which in Molpro is controlled by the keyword DOMOPT = {tight, very tight}, the recovered correlation energy can be increased significantly. This is also shown in Figure 2 as exemplary for the DNA strand made by two guanine-cytosine base pairs. By tightening the aforementioned thresholds, the unaccounted correlation energy decreases from 0.14% to 0.10%. However, by increasing the recovered correlation energy from 99.66 to 99.86% for the tight settings or even 99.90% for the very tight settings, the achieved computational speed-up decreased significantly. Although the regular thresholds show a speed-up factor of 20, gaining 0.20% more correlation energy via the tight settings reduces the speed-up factor to 5. With the very tight settings, it is possible to recover 0.24% more correlation energy compared to the regular thresholds but the computational speed-up reduces to a factor of 4. However, it should be noted that absolute energies are of little interest, and chemical processes are described by relative energies which benefit from error compensation. For those purposes, it has already been shown that the PNO-based methods lead to an accuracy even below 1 kJ mol^–1^.^24,34,39,40^ We will confirm this within the multicomponent context in our model applications.

**Table 2:** System Information on the Guanine-Cytosine Base Pairs and Corresponding Computational Timings for the DF-NEO-MP2 and NEO-PNO-LMP2 Methods^a^

base pairs	1	2	3
basis functions (electronic, nuclear)	(1690, 111)	(3264, 222)	(4838, 333)
fitting basis functions (electronic, nuclear)	(7268, 480)	(14,052, 960)	(20,836, 1440)
occupied orbitals (electronic, nuclear)	(170, 3)	(330, 6)	(490, 9)
average PNO domain size	56.5	51.0	49.3
*t*_CPU_ (DF-NEO-MP2) /s	895.6	21,909.5	160,006.3
*t*_CPU_ (NEO-PNO-LMP2) /s	222.1	1086.4	2734.3
*t*_CPU_ (NEO-PNO-LMP2, electronic–electronic) /s	217.9	1039.8	2492.7
*t*_CPU_ (NEO-PNO-LMP2, electronic–nuclear) /s	4.2	46.5	241.4
*t*_CPU_ (NEO-PNO-LMP2, nuclear–nuclear) /s	0.0	0.1	0.2

a For the domain approximations, the following thresholds were applied: obtained primary PAO domains from IBO charges with a threshold of 0.2, using a connectivity threshold of 2 and a radius of 5 *a*_0_ for the domain extension, an OSV domain occupation number threshold of 10^–9^, a PNO domain occupation number threshold of 10^–8^, and a PNO domain energy threshold of 0.997. The local density fitting connectivity criterion of the domains was set to 2 and the distance criterion to 5 *a*_0_. For the RI approximation, the connectivity criterion of the domains was set to 3 and the distance criterion to 7 *a*_0_. The pair approximations were set to energy thresholds of 10^–4^*E*_h_ for close pairs, 10^–5^*E*_h_ for weak pairs, 10^–6^*E*_h_ for distant pairs, and 10^–7^*E*_h_ for very distant pairs.

**Figure 3:**
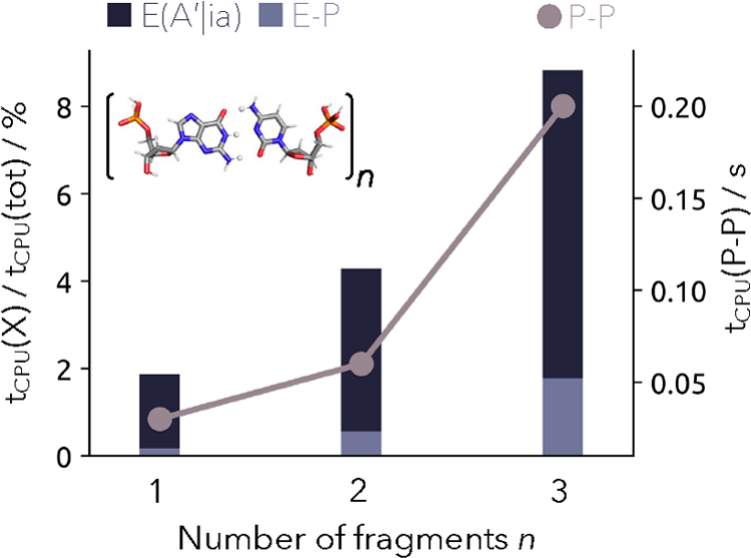
Computational time of the electron–proton correlation is shown as a fraction of the total computational time for the construction of the electronic 3-index integrals E(A′|ia) with the nuclear density fitting basis A′ (dark blue) and the computation and evaluation of *K*_aa′_^ii′^ denoted as E–P (light blue) along with the overall computational time of the proton–proton correlation (light gray).

Before we demonstrate the capabilities of the method, there is one last point to discuss in the general implementation scheme of local approximations within multicomponent methods. As previously discussed in our LDF-NEO-RHF implementation, the major fraction of the computational time arises from the largely delocalized canonical orbitals of the electrons, whereas the nuclei are rather compact and local.^13^ Therefore, we analyzed the timings for individual contributions of the multicomponent correlation method. For this analysis, we compare the NEO-PNO-LMP2 timings of the DNA test systems. Thereby, the most demanding electron–electron interaction is already significantly reduced, as shown above. However, the impact of the electron–proton and proton–proton interactions on the total computational times has not been separately discussed. In Figure 3, we explicitly show their contribution. By increasing the number of quantum protons from 3 to 9, the absolute computational time for the proton–proton correlation rises only from 0.03 to 0.20 s, and therefore, any further local approximation is irrelevant. The electron–proton correlation is computationally much more demanding since it also scales with the number of electrons. As previously mentioned, our implementation makes effective use of the nuclear fitting basis in order to reduce the timings. The fraction of the computational timing for the electron–proton correlation rises from 1.87 to 8.83%, again going from 3 to 9 protons. So even for a large system with 9 quantum protons and 980 electrons, the computational time of the electron–proton correlation part is small compared to the dominating computational time of the electron–electron correlation. By dissecting the computational time of the electron–proton correlation further, it can be observed that the computationally most demanding part is indeed due to the canonical electronic three-index integrals E(A′|ia) within the nuclear fitting basis A′. The integral assembly of *K*_*aa*′_^*ii*′^, which has nominally the higher computational scaling with asymptotically fifth order, only increases from 0.17 to 1.78%. So in general, there is also little gain in introducing local approximations to the nuclei when computing the electron–proton correlation. In order to reduce the computational time in future versions, different strategies can be employed. The first strategy, probably most complex, would be to compute the electron–proton correlation with PNO approximations. However, the simpler and more efficient implementation scheme would be to introduce further and tighter screening procedures which only compute electronic three-index integrals whereby the electronic orbitals ϕ_*i*_ and ϕ_*j*_ are close to the proton center. The third strategy would be to develop a tailored nuclear fitting basis set A′ with fewer functions than the 10s10p10d10f fitting basis set. This would significantly reduce the computational time for the computation of the three-index integrals E(A′|ia). We leave this point for future work and demonstrate in the following the actual capabilities of a composite NEO approach as mentioned earlier, where the level of the electronic–electronic correlation is further increased by explicit correlation and more accurate correlation methods.

### Composite NEO Approach

**Figure 4:**
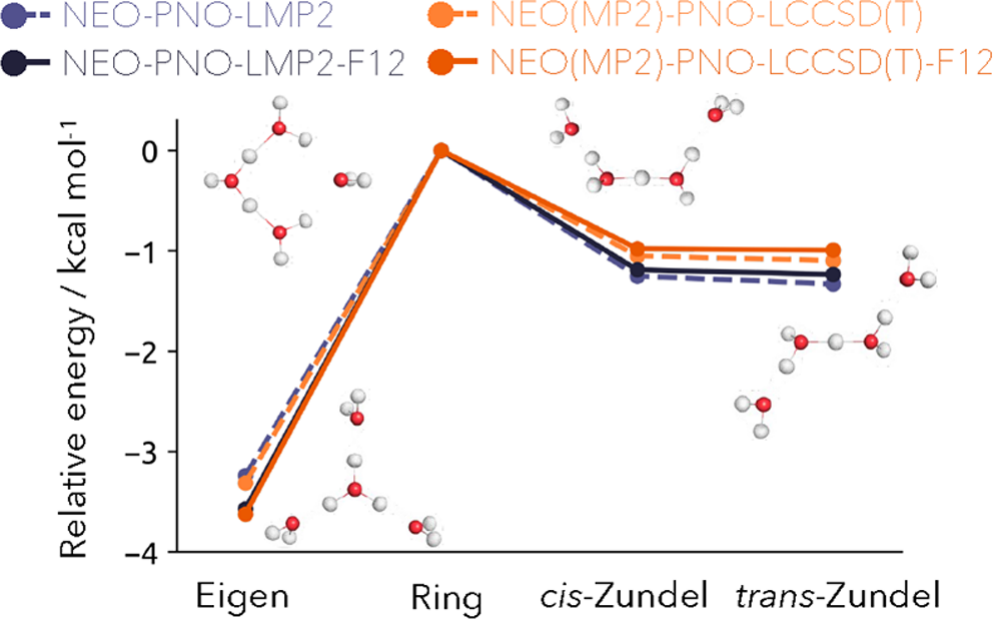
Relative energies of the protonated water tetramers with respect to the ring isomer computed with the NEO-PNO-LMP2 (blue) and NEO(MP2)-PNO-LCCSD(T) (orange) method shown as dashed lines and their explicitly correlated F12 counterpart as solid lines.

First, we benchmark the performance of the two composite NEO variants on one of the most prominent test cases for multicomponent methods: the energetic ordering of protonated water tetramers. The commonly used gold standard for electronic structure calculations, CCSD(T) fails to predict the correct energetic ordering of the four isomers. This can only be restored adequately when anharmonic zero-point vibrations are included or multicomponent methods are employed directly.^22,23^ In our previous work we demonstrated that the correct ordering can be achieved qualitatively by employing CCSD(T) energies with a correction based on the LDF-NEO-RHF reference wave function.^13^ The quantitative results from NEO-PNO-LMP2-F12 and NEO(MP2)-PNO-LCCSD(T)-F12 are shown in Figure 4 together with their regular counterparts. Additionally, values with the Eigen isomer as a reference are provided in Table S1. It should be noted again that within the NEO(MP2)-PNO-LCCSD(T)-F12 the electron–electron correlation is decoupled from the nuclear correlation terms. First of all, we confirm the accuracy of our method for relative energies by comparing the results obtained with NEO-PNO-LMP2 to canonical DF-NEO-MP2 results. The absolute differences are only 0.02, 0.01, and 0.01 kcal mol^–1^ for the Eigen, *cis*-Zundel, and *trans*-Zundel isomers with the Ring structure as reference. As mentioned before, the inclusion of explicit correlation will reduce this domain error further.^33,34^ Noticeably, NEO-PNO-LMP2-F12 and NEO(MP2)-PNO-LCCSD(T)-F12 result in almost the same energy for the Eigen isomer only differing by 0.05 kcal mol^–1^. However, the difference in the energetic ordering for the *cis*-Zundel and *trans*-Zundel is more pronounced with a difference of 0.21 kcal mol^–1^ and 0.24 kcal mol^–1^. As shown by the group of Hammes-Schiffer this difference can be reduced by employing spin-scaling and orbital optimization to the NEO-MP2 method.^23^ If compared to the anharmonic corrected energies obtained by the group of Hammes-Schiffer, the energetic differences to our NEO(MP2)-PNO-LCCSD(T)-F12 method with the Eigen isomer as reference are 0.48, 0.46, 0.27 kcal mol^–1^ for the Ring, *cis*-Zundel and *trans*-Zundel isomer.^22^ Compared to the NEO-CCSD method, the inclusion of explicit correlation and scaled perturbed triple excitations for the electron–electron correlation lowers the energies further by −0.52, −0.24, and −0.23 kcal mol^–1^ of the aforementioned isomers. Moreover, we achieve a similar energetic degeneration of the *cis*-Zundel and *trans*-Zundel isomer.^22,23^

We would like to compare the overall performances of the aforementioned methods with respect to the NEO(MP2)-PNO-LCCSD(T)-F12 method. The corresponding values are given in Table S2. Our previously mentioned PNO-LCCSD(T) + ΔNEO method predicts the correct energetic ordering of the four isomers, but has the highest root-mean-square deviation (RMSD) with 0.84 kcal mol^–1^ of all methods compared.^13^ All isomers are energetically higher in comparison to the NEO(MP2)-PNO-LCCSD(T)-F12 results. By including multicomponent correlation, the RMSD significantly lowers as expected. The NEO-PNO-LMP2 method achieves an RMSD of 0.33 kcal mol^–1^, whereby the Eigen isomer is 0.38 kcal mol^–1^ higher in energy and the *cis*- and *trans*-Zundel isomers are overstabilized by −0.27 and −0.34 kcal mol^–1^, respectively. By including explicit correlation within the NEO-PNO-LMP2-F12 method, the RMSD lowers to 0.19 kcal mol^–1^. This is largely due to the energetic lowering of the Eigen isomer, leading to a deviation of only 0.05 kcal mol^–1^. However, the method overstabilizes the *cis*- and *trans*-Zundel isomers by −0.21 and −0.24 kcal mol^–1^. Compared to the NEO(MP2)-PNO-LCCSD(T) method, which also has an RMSD of 0.19 kcal mol^–1^, the deviation to the Eigen isomer is 0.31 kcal mol^–1^, whereas there is only a small overstabilization for the *cis*- and *trans*-Zundel isomers with −0.07 and −0.10 kcal mol^–1^. Overall, the impact of the explicit correlation is most noticeable for the stability of the Eigen isomer, whereas the energetic order for the *cis*- and *trans*-Zundel isomers is only slightly affected.

It should be noted that slight differences in the energies could arise by using different optimization methods. We optimized the isomers utilizing B2PLYP-D3(BJ) whereas the study of Hammes-Schiffer and co-workers utilized CCSD(T). However, one advantage of multicomponent approaches is the delocalization of nuclei which renders the exact placement of the basis functions and therefore the underlying optimization difference in the quantum mechanically treated nuclei for this system less significant. We will further discuss this aspect in other calculations.

**Figure 5:**
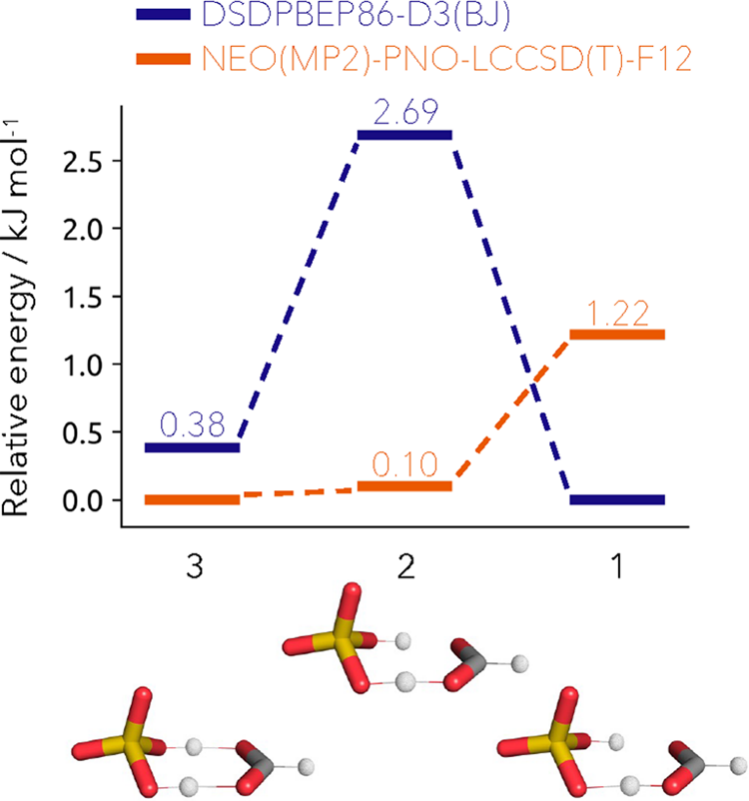
Relative energies of the proton-bound dimer of hydrogen sulfate and formate computed with DSDPBEP86-D3(BJ) (blue) and NEO(MP2)-PNO-LCCSD(T)-F12 (orange).

As another benchmark system, we employ the proton-bound dimer of hydrogen sulfate and formate. This is a challenging case for electronic structure methods since it exhibits a shallow potential energy surface with zero-point energy in the order of the proton translocation.^54^ Therefore, accurate methods going beyond the Born–Oppenheimer approximation have to be employed to capture nuclear quantum effects and restore a quantitatively accurate description of the system. Recently, path-integral molecular dynamic (PIMD) studies were carried out on the system within a temperature range from 100 to 300 K.^65^ In order to close the gap to the measurement at cryogenic temperature, multicomponent methods such as our NEO(MP2)-PNO-LCCSD(T)-F12 approach can be utilized. The great advantage of such approaches is that nuclear delocalization and anharmonic zero-point energies of quantum nuclei are inherently included. This is computationally much more efficient and robust.^12^ Thomas et al. compared different and commonly applied electronic structure methods and found a large spread between the obtained results.^54^ In Figure 5, we show exemplary results for those methods, the DSDPBEP86-D3(BJ) results together with our NEO(MP2)-PNO-LCCSD(T)-F12 energies computed on their structures. The double-hybrid DSDPBEP86-D3(BJ) method predicts a barrier with transition structure **2** connecting global minimum structure **1** to slightly higher local minimum structure **3**. The multicomponent picture of the potential energy surface is entirely different. Under NEO, the global minimum is structure **3** followed by slightly higher but almost energetically degenerate structure **2**. Structure **1** is the most unstable, forming a shallow potential surface. This potential falls in agreement with the PIMD results reported in the literature. In the results from Udagawa et al., it was observed that at a high temperature of 300 K, the picture is consistent with the double-hybrid DSDPBEP86-D3(BJ) potential surface (predominance of structure **1**, followed by **3**). However, by lowering the temperature down to 100 K, the probability of observing structure **1** lowers, while the populations of structures **2** and **3** increase, consistent with a shallow potential energy surface.^65^ This is in line with our NEO(MP2)-PNO-LCCSD(T)-F12 results, which are representative of 0 K. Taking a close look at the respective structures, this trend can be further explained. While structure **1** only has one shared proton centered toward the formate, structure **2** exhibits a perfectly shared proton between the hydrogen sulfate and the formate and the energetically lowest structure **3** has two shared protons both centered toward the hydrogen sulfate. In future work, it would be interesting to optimize these structures within a multicomponent framework and predict the actual vibrational frequencies accordingly. This would allow us to compare to the experimental values and benchmark the approach.^68^ This could be, for example, achieved by using the NEO-DFT(V) method developed within the group of Hammes-Schiffer.^69^ With this objective in mind, we are currently developing a computationally efficient protocol of the NEO-DFT and NEO-TDDFT methods within Molpro by employing local density fitting approximations.

**Figure 6:**
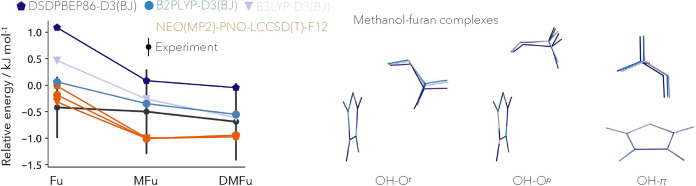
Left: Relative energies of the energetically more stable OH–O bound methanol complexes with furan (Fu), 2-methylfuran (MFu), and 2,5-dimethylfuran (DMFu) computed with DSDPBEP86-D3(BJ) (dark blue), B2PLYP-D3(BJ) (blue), B3LYP-D3(BJ) (light blue), and NEO(MP2)-PNO-LCCSD(T)-F12 energies on the respective structures (orange) with respect to the OH–π bounded complex. The experimental estimates and error bars are taken from Gottschalk et al.^66,67^ Right: Respective conformers of methanol-furan optimized with DSDPBEP86-D3(BJ) (dark blue), B2PLYP-D3(BJ) (blue), and B3LYP-D3(BJ) (light blue), MFu and DMFu conformers shown in Figure S1.

In the following, we aim to benchmark our NEO(MP2)-PNO-LCCSD(T)-F12 method with other commonly applied electronic structure methods and compare it to experimental data. Therefore, we employ as test systems the hydrogen-bound methanol complexes with furan (Fu), 2-methylfuran (MFu), and 2,5-dimethylfuran (DMFu) reference systems. Those systems were already thoroughly investigated by both theory and experiment as part of a blind challenge.^66,67^ We optimized the OH–O^*t*^, OH–O^*p*^, and OH–π structures of the respective dimers with DSDPBEP86-D3(BJ), B2PLYP-D3(BJ), and B3LYP-D3(BJ). The obtained structures are shown in Figures 6 and S1. The zero-point-corrected energy differences between the most stable OH–O bound structures with respect to the OH–π structure are shown in Figure 6 together with the respective NEO(MP2)-PNO-LCCSD(T)-F12 energies. Thereby, the hydrogen stretching frequency of the quantum mechanically treated hydrogen of the hydroxyl moiety was removed from the zero-point energy to avoid double counting since this contribution is intrinsically included in the NEO energy. The obtained energies are displayed in Table S3. We start by discussing the most noticeable feature of the NEO results shown in the graph compared to the other methods. Although the individual structures differ quite substantially from each other by allowing the most important hydrogen to delocalize, the difference in energies narrows down. Therefore, NEO(MP2)-PNO-LCCSD(T)-F12 can be used to obtain reliable and robust results, relatively independent of the underlying optimization method. Moreover, independently of the used optimization method the NEO(MP2)-PNO-LCCSD(T)-F12 method predicts, as the only method within the reference set, that the OH–O^*t*^ structure is energetically favorable over the OH–O^*p*^ and OH–π structures for the tested complexes. This was already suggested by experiment and later on explicitly confirmed for the methanol-furan dimer by rotational spectroscopy.^66,67^ In contrast, every other method employed in this work fails to predict the physically correct trend and instead favors the OH–π structure for the methanol-furan dimer. One more point to note is that anharmonic corrections applied by multiple groups participating in the blind challenge also lead to inconsistent results regarding the stability of the conformers. This was, in fact, the most critical point of the entire challenge. This highlights the robustness of the NEO(MP2)-PNO-LCCSD(T)-F12 ansatz, given that all obtained NEO results are within the experimentally determined error bar. The same is only achieved by B2PLYP, however predicting the wrong stability of the furan methanol dimer. Overall, the NEO(MP2)-PNO-LCCSD(T)-F12 method provides a computationally efficient, very accurate, and robust approach to tackle complex problems where NQEs are relevant for chemical selectivity.

## Conclusions

In this work, we not only presented an efficient implementation of the density-fitted NEO-MP2 method for small to midsize systems but also for the first time introduced local approximations within a multicomponent correlation treatment. We demonstrated that with our approach, large systems can be computed at a fraction of the computational cost of the canonical counterpart. As such, correlation energies including NQEs can be obtained in an efficient and accurate manner paving the way for their routine application in a suite of challenges, from ab initio drug development to material design.^70−73^ Our composite approach successfully describes systems where regular electronic structure methods are qualitatively and quantitatively incorrect. In future work, we will continue improving our composite method accuracy-wise. There are different points that we should address in the future. One is to improve the description of electronic–nuclear correlation, which is known to significantly affect the locality of the proton density.^37,74^ NEO-MP2 is the first step in the ladder for wave function correlation but lacks higher-order effects. Another issue is that the residuals for the electronic–nuclear correlation do not include a coupling to electronic amplitudes. This can be included by other correlation treatments such as NEO-CCSD^74^ or NEO-OOMP2,^75^ both of which agree well with reference densities for model systems. Albeit the impact on protonic densities is documented, how electronic–nuclear correlation affects the energetics and properties of molecular systems is still somewhat unclear. The reason for this is the limited number of applications making use of a multicomponent framework to date.

We also plan to extend the use of density fitting for multicomponent methods, with a focus on implementing a local density-fitted NEO-DFT method as well as a density-fitted version of the NEO-TDDFT method.^29−31,76,77^ Both are needed within the NEO-DFT(V) method, which provides accurate vibrational frequencies with multicomponent methods.^69^ Besides the implementation work, tailoring nuclear auxiliary fitting basis sets for the well-established nuclear PB basis sets will further increase the accuracy and lower the computational costs of density-fitted approaches.^41^

## Data Availability

Structural information and energies are available free of charge on GRO.data (10.25625/0XAFKE).

## Supporting Information Available

Relative energies of the protonated water tetramers with respect to the energetically most stable Eigen isomer (Table S1), deviations of the relative energies of the protonated water tetramers with respect to the ring isomer in relation to the NEO(MP2)-PNO-LCCSD(T)-F12 results for the respective NEO methods employed and their root-mean-square deviation (Table S2), relative energies of the methanol complexes with the furan derivatives for both OH–O bound complexes with respect to the OH–π complex (Table S3), and conformers of methanol-2-methylfuran and methanol-2,5-dimethylfuran complexes (Figure S1) (PDF)
