# Supplementary material for: Local Electronic Correlation in Multicomponent Møller–Plesset Perturbation Theory
Source: J Chem Theory Comput. 2024 Nov 8;20(22):9928–38. doi: 10.1021/acs.jctc.4c01059 (PMC11603598; doi:10.1021/acs.jctc.4c01059)
Supplement: Supplementary file 1 — ct4c01059_si_001.pdf [file ct4c01059_si_001.pdf]

Supporting Information: Local electronic correlation in multicomponent Møller-Plesset perturbation theory

Lukas Hasecke* and Ricardo A. Mata*

Institute of Physical Chemistry, University of Göttingen, Tammannstrasse 6, 37077, Göttingen, Germany

E-mail: lhaseck@gwdg.de; rmata@gwdg.de

Table S 1: Relative energies of the protonated water tetramers with respect to the energetically most stable Eigen isomer given in kcal mol^{-1} for the respective NEO correlation methods.

Method	Ring	*cis*-Zundel	*trans*-Zundel
DF-NEO-MP2	3.26	2.00	1.92
NEO-PNO-LMP2	3.24	1.99	1.91
NEO-PNO-LMP2-F12	3.57	2.38	2.34
NEO(MP2)-PNO-LCCSD(T)	3.31	2.26	2.22
NEO(MP2)-PNO-LCCSD(T)-F12	3.62	2.64	2.63

Table S 2: Deviations of the relative energies of the protonated water tetramers with respect to the Ring isomer in relation to the NEO(MP2)-PNO-LCCSD(T)-F12 results given in kcal mol^{-1} for the respective NEO methods including the root mean square deviation (RMSD) in kcal mol^{-1}.

Method	Eigen	*cis*-Zundel	*trans*-Zundel	RMSD
PNO-LCCSD(T)+Δ NEO	1.35	0.32	0.44	0.84
NEO-PNO-LMP2	0.38	-0.27	-0.34	0.33
NEO-PNO-LMP2-F12	0.05	-0.21	-0.24	0.19
NEO(MP2)-PNO-LCCSD(T)	0.31	-0.07	-0.10	0.19

Table S 3: Relative energies of the methanol complexes with the furan derivatives for both OH-O bound complexes with respect to the OH-π complex computed with DSDPBEP86-D3(BJ), B2PLYP-D3(BJ) and B3LYP-D3(BJ) and NEO(MP2)-PNO-LCCSD(T)-F12 energies on the respective structures in kJ mol^{-1}.

Complex	Furan	2-methylfuran	2,5-dimethylfuran
DSDPBEP86-D3(BJ)			
O^t	1.16	0.08	-0.05
O^p	1.09	2.46	2.64
NEO(MP2)-PNO-LCCSD(T)-F12//DSDPBEP86-D3(BJ)			
O^t	-0.02	-1.00	-0.98
O^p	0.02	1.16	1.52
B2PLYP-D3(BJ)			
O^t	0.51	-0.35	-0.55
O^p	0.06	1.23	1.20
NEO(MP2)-PNO-LCCSD(T)-F12//B2PLYP-D3(BJ)			
O^t	-0.18	-1.01	-0.94
O^p	0.00	1.07	1.50
B3LYP-D3(BJ)			
O^t	0.73	-0.26	-0.61
O^p	0.47	1.12	1.00
NEO(MP2)-PNO-LCCSD(T)-F12//B3LYP-D3(BJ)			
O^t	-0.32	-1.00	-0.98
O^p	-0.15	0.98	1.31

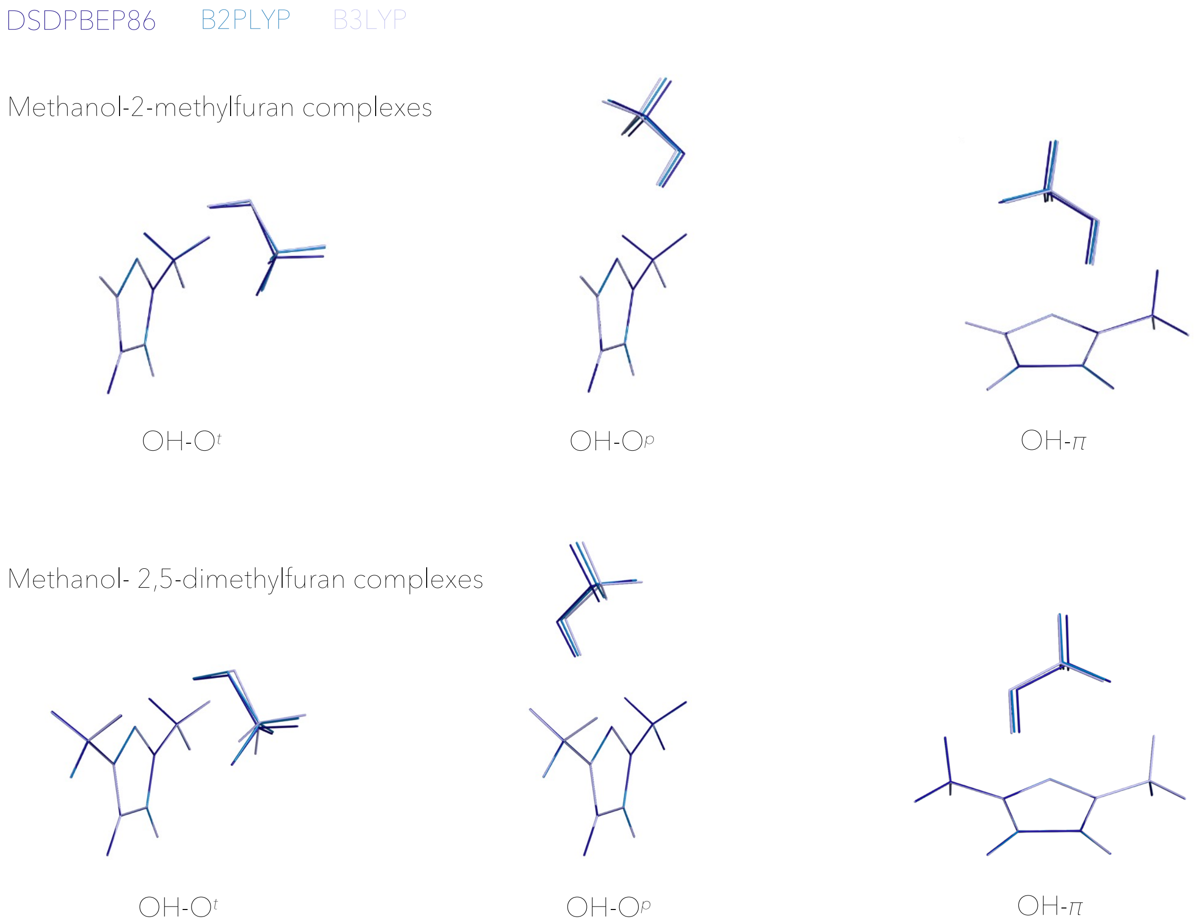

Figure S1: Conformers of methanol-2-methylfuran and methanol-2,5-dimethylfuran complexes optimized with DSDPBEP86-D3(BJ) (dark blue), B2PLYP-D3(BJ) (blue) and B3LYP-D3(BJ) (ligth blue).
